# Kératoses séborrhéiques: une localisation inhabituelle

**DOI:** 10.11604/pamj.2017.27.53.10913

**Published:** 2017-05-24

**Authors:** Hasnaa Zaouri, Baderddine Hassam

**Affiliations:** 1 Service de Dermatologie-Vénéréologie, Centre Hospitalier Universitaire Avicenne, Université Mohammed V, Rabat, Maroc

**Keywords:** Kératoses séborrhéiques, verge, génitale, Seborrheic keratoses, penis, tumors

## Image en médecine

Les kératoses séborrhéiques (KS) sont des tumeurs épithéliales bénignes fréquentes. Elles se développent préférentiellement dans les zones séborrhéiques,notamment le visage et le tronc, rarement au niveau génital.Nous rapportons le cas d’un patient âgé de 41 ans, sans antécédents pathologiques notables, consultait pour des lésions pubiennes évoluant depuis trois ans. L'examen clinique retrouvait des lésions noirâtres à surface kératosique, cérébriformes au niveau de la verge. La biopsie cutanée était en faveur d’une kératose séborrhéique. Un traitement par électrocoagulation-curetage a été réalisé.Il existe deux théories expliquant le mécanisme de formation des KS. La première, émet l’hypothèse que les KS sont la résultante d’une prolifération de kératinocytes aboutissant à une inhibition de l’apoptose et l’expression de la survivine. D'après la seconde, les KS seraient le résultat d’une accumulation de kératinocytes sénescents avec une surexpression de la protéine p16. Dans le cas des KS génitales, le rôle du frottement chronique et de l’HPV a été suspecté. Le traitement repose sur l'exérèse chirurgicale, le curetage, la cryothérapie par azote liquide, l'application d'acide trichloroacétique, l'électrosection ou l’ablation par laser CO2. Si les KS ne posent pas a prioride problèmes de santé majeurs, leur localisation au niveau de la région génitale peut influencer négativement la vie sexuelle du patient, d’où l’intérêt d’une prise en charge précoce.

**Figure 1:**
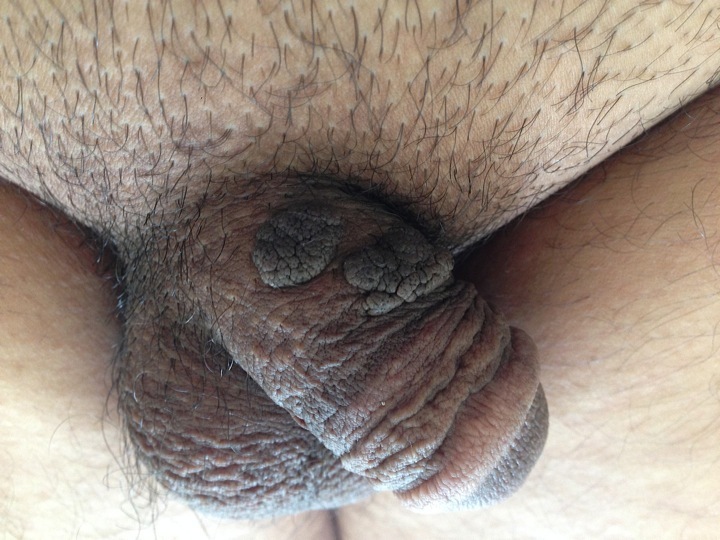
Kératoses séborrhéiques de la verge

